# Understanding Structural Violence in Community Violence Intervention (CVI): A Multi-City Qualitative Analysis of Practitioner Perspectives

**DOI:** 10.1177/00469580251376234

**Published:** 2025-09-16

**Authors:** Asia S. Ivey, Julia J. Lund, Amanda J. Aubel, Shani A. L. Buggs

**Affiliations:** 1 Centers for Violence Prevention, University of California, Davis, Sacramento, CA , USA

**Keywords:** structural violence, community violence, CVI practitioners, training and professional development, community-based violence intervention

## Abstract

The discourse on community violence has expanded over the years, shifting from a focus on interpersonal physical harm to a broader understanding that includes systemic and structural harm. Structural violence, characterized by institutionalized inequities in health, education, and generational wealth, disproportionately impacts marginalized communities and reflects deliberate systems of oppression designed to maintain power imbalances. In community-based violence intervention and prevention (CVIP), identifying how harm can be systematically perpetuated is critical for developing and advancing structurally grounded evaluative measures and training strategies for practitioners. This qualitative study involved interviews and focus groups with community violence intervention (CVI) practitioners (N = 45) from Sacramento, Milwaukee, and Baltimore. We analyzed participants’ narratives to explore their understandings of the root causes of community-based firearm violence, with particular attention to the core tenets of structural violence: power, marginalization, oppression, adversity, and trauma. Findings revealed that CVI practitioners hold varying levels of structural violence expertise, ranging from individual-level explanations of violence to critical accounts of how systemic forces cultivate and reproduce structural harm. Participants discussed how government divestment, institutional neglect, and collective and vicarious trauma shape the conditions contributing to community violence. Their reflections underscore the need for standardized training and professional development that embeds structural frameworks into CVIP operations and program evaluations. As key actors in CVIP, CVI practitioners must be equipped with the knowledge and skills to address the structural drivers of community violence. Investing in their capacity for research and advocacy will strengthen the field’s effectiveness, scale, and legitimacy in preventing community-based firearm violence through structurally informed practice and evaluation.

**Highlights**

● Applies structural violence as a critical theory for understanding the root causes of community-based firearm violence.

● Examines the understanding of structural violence among CVI practitioners.

● Utilizes qualitative data with 45 CVI practitioners across Sacramento, Milwaukee, and Baltimore.

● Demonstrates how practitioners interpret the root causes of violence, ranging from individual to systemic perspectives.

● Recommends the development of standardized training and evaluation methods that integrate structural analysis in CVI practice.

## Introduction

The discourse surrounding community violence has evolved significantly over the years, expanding beyond interpersonal physical violence to encompass the systemic and structural forces that perpetuate harm on a broader scale.^1^ This evolution, integral to understanding the complex nuances of community-based violence, is primarily due to an increasing number of researchers, practitioners, and community organizers reconsidering traditional constructions of community-based violence and, thus, approaches to community violence intervention and prevention (CVIP).^2^ Structural analyses of community-based violence have illuminated how significant harms have been enacted upon marginalized groups and persisted in forms historically unacknowledged and, thereby, unchallenged.^3,4^ While acts of physical violence have often dominated and continue to overwhelm mainstream narratives, a more insidious form of violence, structural violence, has covertly erected barriers that disproportionately hinder Black and Brown communities from thriving.^5-8^ The field of community violence intervention (CVI) addresses violence as a public health issue and mitigates future harm by addressing its immediate and root causes. Consequently, the evaluation of CVI organizations and their prevention efforts must account for the structural and systematic proliferation of violence, which has proven challenging to measure and apply in practice.

Reconceptualizing a hegemonic phenomenon such as violence necessitates reexamining the notion of a singular perpetrator of harm and acknowledging the latent forces that shape conditions for safety or insecurity. Parsons emphasizes the need “to de-emphasize the role of intentionality in the cause and propagation of harms” (p. 174)^9^ and expand the understanding of violence beyond its immediate manifestations. Moreover, Galtung’s structural violence framework broadens the conceptualizations of violence to encompass the reproduction of harm, its consequences, and the inequities perpetuated due to its occurrence.^9^ Analyzing violence through this nuanced and contextualized lens involves focusing on its impact, addressing its avoidable nature, and ensuring those impacted are protected from recurring harm. This expanded perspective is critical for advancing CVIP efforts and highlights the importance of recognizing the perpetuation of veiled harm.

Structural violence is challenging to measure and evaluate; however, researchers have illuminated core elements that are integral to how structural violence operates and reproduces harmful inequities. Jackson and Sadler’s literature review revealed five attributes central to structural violence,^10^ power,^11,12^ marginalization,^13,14^ oppression,^15,16^ adversity,^14,17^ and trauma.^11,18^ Power is arguably the most critical aspect of structural violence, as it is inextricably linked to structure and agency and is responsible for the inequitable distribution of resources, influence, and harm.^12^ These core attributes establish the modus operandi of structural violence^11^ and should be examined relationally, with attention to their consequential effects on health inequities, injustice, and social dysregulation.^10^

Further, as identified by Jackson and Sadler, the processes of embodiment and dispossession cyclically cooperate to maintain structural violence. Embodiment is the process by which societal values, informed by prejudice and discrimination, are embedded and personified in daily norms, policies, and practices.^11^ Dispossession captures how the lived effects of embodiment intensify the exclusion of those considered inferior, such as through carceral system involvement or cycles of intergenerational poverty. This occurs because the policies and practices designed to govern society instead leave marginalized groups disproportionately vulnerable from the beginning.^10,14^ Although the field of CVIP recognizes the value of examining the social and environmental factors that contribute to community-based violence, program evaluations continue to lack the appropriate structural measures to accurately examine programmatic operations and successes.^19^

When CVIP program evaluations are grounded structurally, fundamental organizational elements that are often overlooked or unquantifiable can be operationalized and examined, such as the roles, insights, and contributions of program staff. As key actors in the field of CVIP, it is critical to understand the praxis, or theory of action, of community violence intervention (CVI) professionals, as it informs their understanding and approaches to the work.^19^ CVI professionals, committed to addressing the harms of systems and trauma through outreach and intervention efforts, are uniquely positioned to engage with those most at risk of violence and bring invaluable trust and credibility to their role in mitigating violence.^20^ However, CVI professionals differ in their conceptualizations of violence and its root causes. Given how their theory of action can inform how they engage with clients and envision programmatic success,^19^ it is vital that evaluative measures account not only for CVI professionals’ unique experiences and proximity, but also for their level of structural competency.

According to researchers, the ongoing proximity of CVI professionals to those at risk of violence necessitates advanced skills and training in violence prevention strategies.^21^ Knox and Spivak assert that practitioners who work in youth violence tend to operate at three levels: *generalist, specialist*, and *scholar. Generalist-level* competency in structural violence includes understanding the various causes of youth violence and possessing an attitude that accounts for its social and environmental catalysts. Most CVI professionals operate at the *specialist level*, where their responsibilities often include working closely with clients most at risk, and their families, assessing needs, developing safety plans, and connecting clients with critical resources.^21^ However, researchers recommend that individuals with specialized and ongoing roles in violence prevention, like CVI practitioners, obtain *scholar-level* competency, which requires more in-depth engagement with structural factors that inform community-based violence, such as policy, systems, and societal narratives. Achieving scholar-level competency across domains involves engaging in activities that include establishing working groups in the community, researching causes of community-based violence, evaluating the effectiveness of interventions, and advocating for CVIP at local and state levels.^21^

CVI professionals demonstrate a strong commitment to enhancing their skills and advancing their professional development; however, their progress in this area is often hindered by a lack of accessible opportunities and the absence of standardized credentials to signal topic area expertise.^22^ Considering how interpersonal conceptions of violence failed to acknowledge how structural forces also perpetuate harm, it is valuable to consider what might be overlooked when CVI professionals lack a robust structural perspective on violence due to limited advanced training and professional development opportunities to move beyond their demonstrative efforts and into more critical evaluative methods. According to Jackson and Sadler, structural violence frameworks are a medium through which researchers, educators, practitioners, and community organizers can become advocates and leaders in the fight against macrosystemic oppression.^10^ As such, the field of CVIP should recognize structural violence competency as an integral component of CVI professionals’ training and advancement.

The analysis presented in this work is part of a larger research study that sought to broaden the field’s understanding of CVI professionals and their experiences on the frontlines. The interview and focus group study involved gathering the perspectives of CVI practitioners on the drivers and mitigators of violence, workplace challenges, and needs concerning themselves and their clients. This work builds upon those efforts, continuing to center CVI professionals as experts in the field while contextualizing their insights within structurally grounded theoretical frameworks. By illuminating the structural perspectives of CVI professionals, this paper highlights the need for the evaluative and training domains of CVIP to address the root causes of community-based violence to fully realize the field’s potential.

## Methods

The data employed for this study were gathered during an extensive qualitative study, which has been previously described.^23^ Participants (N = 45) were recruited from Sacramento, California; Milwaukee, Wisconsin; and Baltimore, Maryland (2022-2023), to capture diverse perspectives on the distinct dynamics of violence in each city, as well as the structural factors that commonly drive community unrest. The cities were chosen due to their higher-than-average violence rates and significant local investments in their respective CVIP efforts. The senior author, Dr. Shani Buggs, leveraged her existing connections for study recruitment. Convenience and purposive sampling methods were utilized, ensuring each participant possessed the necessary experiential and content expertise.^23,24^

Prior to data collection, each participant was presented with a verbal consent document, which was read aloud and explained in full. Participants were given an opportunity to ask questions and were asked to verbally confirm their willingness to participate before the interview or focus group began. This process was approved by the University of California, Davis Institutional Review Board, which granted exempt status based on *Exemption Termination HRP-312*, due to the minimal-risk nature of the research and absence of identifiable information. This study was conducted and reported in accordance with the COREQ (Consolidated Criteria for Reporting Qualitative Research) guidelines for qualitative research involving interviews and focus groups (Supplemental Material).^25^

Interviews and focus groups followed a semi-structured guide and were audio-recorded, transcribed using Rev.com, and then thematically coded. For this paper, we inductively focused on a subset of codes most relevant to CVI professionals’ structural perspectives concerning violence due to emerging variations in narratives concerning the root causes of violence. The analysis involved reviewing relevant excerpts that directly (semantic-level analysis) or indirectly (latent-level analysis) described how the core tenets of structural violence (power, marginalization, oppression, adversity, and trauma) or how the processes that maintain structural violence (embodiment and dispossession) contribute to community-based firearm violence. To maintain readability and participant confidentiality, quotations were minimally edited for clarity (e.g., removal of filler words and potentially identifiable details) without altering their core meaning.

## Findings

The study’s findings illuminated themes related to CVI professionals’ structural understandings and the gaps in training on structural frameworks. While CVI professionals did not explicitly identify *structural violence* as a concern for their clients, they articulated the prevalence of systematic power imbalances and generational trauma, and shared examples of marginalization, oppression, and adversity experienced by the clients and communities with whom they engage. Across interviews and focus groups, CVI professionals explained how the manifestations of structural violence are evident in the lack of access to adequate jobs, excessive access to drugs, firearms, and ammunition, insufficient opportunities for upward mobility, heavy police surveillance, and a lack of initiative from the government to ensure that existing resources are more accessible for those at highest risk of violence involvement. While their their lived and professional experiences guide their structural praxis, advanced skills training that strengthens and leverages this specialized knowledge base for research and advocacy will further enrich and legitimize the field of CVIP.

### Structural Violence Perspectives of CVI Practitioners

Structural violence competency exists on a spectrum; thus, there is diversity in CVI professionals’ perspectives on the root causes of community-based firearm violence. For instance, a CVI professional expressed, *“. . .We don’t need no guns right now. . .as a whole, in the condition of our people, we don’t need no guns, ‘cause clearly. . .we don’t do the right things with them. . .we just killing each other with them.”* While firearm access is a structural issue, it is widely discussed as a matter of individual responsibility and moral character, a narrative CVI professionals are not immune to. However, as illustrated by another practitioner with a history of violence involvement, gaining education and awareness can shape behaviors and prevent cycles of violence. They explained that learning about historical, systemic forces, such as the ghettoization of Black communities and broader patterns of oppression, influenced their actions and worldview. In recognizing these structural harms, they gained critical insights into how the integration of power and psychosocial manipulation perpetuates cycles of harm, referring to a “wise enemy” as a powerful systemic force designed to maintain inequity.

If I got a gun, and you can help me to understand why I got this gun, how it ended up in my community. . .Once I understood everything, I stopped doing all that kind of stuff. Once I got the true understanding of what was going on, and why we was doing this, why we was living the life that we was living, how we ended up in the ghetto, the unique experience that Black people came through in America, what we was before we was in America. . . that was enough to make me change. That was enough to make me realize, like, ‘Okay. I was a part of something.’ Like, we have a very wise enemy, and they know how to make you a part of something or make you do the wrong thing, thinking you’re doing the right thing.

This reflection illustrates the transformative potential of education as a tool of empowerment and resistance against systemic oppression. Connecting individual experiences and actions to macrosystemic dynamics, such as power and oppression, can inspire critical consciousness and behavior change.

Another CVI professional shared how their examination of the social engineering within systems of power, across racial and national boundaries, revealed how firearm access is an issue of systematic manipulation, where marginalized and divested communities are disproportionately harmed by policies and practices created to engender cycles of violence.

I can also look into the white community, or another country, and look at their gun violence problems and say, ‘Well, wow. They’re not killing each other at the rate we are. Why?’ And you’ll find out that they don’t have an enemy who is putting the guns [in their communities] and then influencing [them] to kill each other. . .Giving you drugs and giving you guns and influencing you to kill each other, socially engineering you. . .The reality is America has more gun violence than 99% of the world. [T]here’s more guns here than people. So that [should] tell you something. And the guns are concentrated in our communities for a reason.

This CVI professional attributes the concentration of firearms in underserved areas to government disinvestment, deliberate neglect, and coordinated harm, revealing how structural inequities sustain the marginalization and insecurity that contribute to violence

Through policy loopholes and inconsistent state regulations on sales, transfers, ownership, and lethality, firearm manufacturers can flood the market with military-style firearms, accessories, and ammunition disproportionately impacting low-income, Black and Brown communities.^26,27^ Despite this systematic influx of weapons into communitieswith limited protective resources,^28,29^ Black and Brown individuals are disproportionately arrested for illicit possession of firearms.^30^ These patterns reinforce embodied stereotypes of inherent criminality and perpetuate cycles of structural violence, where marginalized groups bear the burden of conditions they did not create, yet are forced to navigate.

When we was coming up, we didn’t have 30. . .50. . .or 100 shots in this AR. You know what I’m saying? And I believe that [is] more reason for us to get involved with our youth. . .’cause those people are allowing those guns, and those clips, to flood our city. So, we fighting the war with the people, but who is fighting the war to keep those guns out of our community? You know what I’m saying? . . .So, I think a lot of this plays back to the government or who is in charge of allowing these manufacturers to [send] the guns into our neighborhoods. . .and they’ve been doing it for years. And they [are] allowing them to get licensed to do this. . .

CVI professionals are frustrated by the unfettered proliferation of high-capacity firearms and the systemic failure to regulate their access and distribution. Drawing from lived experience, they underscore how the increasing availability of firearms and extended magazines has exacerbated violence in their communities. In highlighting the role of firearm manufacturers and policymakers in perpetuating these conditions, the CVI professional calls attention to a significant structural dimension (e.g., manufacturers and regulatory bodies) of firearm violence often overlooked in both public discourse and the evaluation of CVIP programs’ role in reducing community-based firearm violence.

### Embodiment and Dispossession

In their own words, CVI professionals described how the processes of embodiment and dispossession cyclically maintain a structurally violent system. For example, this CVI professional’s sentiments reflect how institutional neglect embodies societal and systemic indifference:

“. . .I don’t know if the city cares enough. Because. . .when things only impact poor people, then it’s like, ‘What? Like, why should it matter?’” I think. . . [the city] has a lot of resources, but the resources are not always accessible. A lot of folks who are impacted by gun violence in [the city] also have, generations of folks who have been impacted by mass incarceration, generations of poverty, generations of domestic violence, generations of trauma. . .

The compounded harm reveals how racialized belief systems of inferiority manifested through policy, practice, and status quo maintenance can influence generational outcomes. When inequity is embedded in resource distribution and institutional investment, structural violence becomes a physical condition of life for marginalized communities, as policies shaped by embodied prejudices reflect, produce, and sustain societal inequities. Thus, disrupting these cycles requires acknowledging and dismantling the underlying belief systems deeply embedded within institutional practices, a long-term process that often extends beyond the scope of traditional evaluation measures in CVIP and necessitates a structurally grounded evaluative approach.

Those in power who dictate resource distribution contribute to victim blaming and the justification of inaction when they possess and reinforce attitudes like “. . .*he had it coming*. . .” a commonly held perspective among many policymakers, according to a CVI expert. This perspective is often reflected in media narratives that couple violent victimization reporting with criminal histories or otherwise signal the devaluation of the lives of Black and Brown victims. These embodied belief systems guide resource distribution and policy priorities, privileging some groups while marginalizing others. The pervasive notion that low-income Black and Brown communities are “less deserving” of critical resources does more than shape attitudes; rather it becomes a tangible reality, dictating who has access to health, safety, and opportunity.

Thelack of investment in the development, implementation, and political will associated with CVIP programs has hindered their progress and directly impacted their ability to succeed.^19,22,31^ CVI professionals in our study recognized how indifference toward CVIP is a systemic issue occurring at all levels of government and permeates the institutions CVIP programs depend on for success, such as healthcare, education, and the legal justice system. This neglect is reinforced by societal narratives that frame community-based violence as isolated events, rather than as outcomes of broader structural conditions.

. . .[W]hat’s the point of working in this if things will never change? On all levels, from federal to state to local, it can get very disheartening just to see how it’s always overlooked. And people don’t really think about community violence as. . .a public health issue. They just read the captions or. . .headlines and just see it as ‘Somebody got upset today.’ That’s always very heavy to just carry with you in this line of work, knowing that there are very, very few people who believe in what you do. From the healthcare system to the legal system to even. . .the community. . .[A]nd because community violence is overlooked and not understood for what it is, there’s not enough resources.

This reflection demonstrates how the embodiment of harmful stereotypes contributes to the diminishment of community violence as a legitimate public health concern. Rather than addressing the root causes of community-based firearm violence, narratives shaped by racial bias vilify individuals and communities most at risk, framing violence as a personal failure rather than the outcome of structural harm. Moreover, the institutional systems that propagate harmful narratives cultivate a culture of fear and self-defense that propagates cycles of violence. Without specifically naming the process of embodiment, another CVI professional captured the psychological impact of these structural forces:

. . .[T]o perceive people and things as dangerous, you get stuck in a mode where you’re on defense all the time. . .So when you have a whole community of people who are told, y’all are dangerous and y’all just savaging, y’all do whatever. Then, you start to internalize that and look at each other that way; your reaction to different situations is gonna be a lot of times, I would say, extreme, and it won’t make sense to other people.

This insight reveals how harmful messaging and generational trauma shape identity and behavior. The internalized shame and mistrust that arises from a history of public discourse characterizing certain groups as inherently violentcreates profound inner conflict.^32-34^ The internalized stigma shapes interactions within communities and with external entities.^35,36^

Over time, dispossession—the process of reinforcing stratified hierarchies based on embodied beliefs—can leave individuals and their communities caught in cycles of structural harm. Generations of systematic indifference, neglect, and inequity have cultivated a pervasive mistrust of institutions among communities disproportionately impacted by violence. This poses a challenge for CVIP organizations, whose work requires collaboration with institutions that have engendered mistrust. Consequently, the perception that CVI practitioners are merely extensions of these systems that have historically abandoned or harmed Black and Brown communities may trigger suspicion among clients.

There’s a lack of trust in systems. . .I think our people have a unique situation in America, and because of our unique situation, we do not trust people who come from systems. It don’t matter what system (laughs) [you’re] from; we just don’t trust them. [S]o therefore, they don’t know that we come from the same backgrounds as them. . .To them, we just somebody else from the system. . .‘What, do they want to know my situation? Like, what are you really trying to find out?’ So I think that’s really what it is. . .and that don’t got nothing to do with us. That got to do with the people that came before us.

CVI professionals must navigate a challenging dilemma created by the deeply entrenched mistrust of the systems that shape society. Although clients may appear resistant to interventions or unmotivated to change their lifestyles, their hesitation may more so reflect a survival mechanism shaped by a legacy of institutional failure; as one CVI specialist stated, *“. . .[W]e’re dealing with young men that are heavily traumatized. . .so they’re skeptical when [we] come to the door. They always associate us with law enforcement. . .”* For prevention efforts to be successful, it is essential for CVI professionals to recognize mistrust as a rational response to trauma and generational harm rather than a lack of motivation. This shift in perspective centralizes the structural and historical conditions that inform their skepticism rather than blaming clients for their hesitancy. Although CVI professionals may learn these nuances on the job, formal ongoing training could ensure targeted skill development in structural competency, particularly with regards to instutitional legacies of oppression.

### Advancing Skill Training and Professional Development

. . .I think street outreach workers deserve and need more training. . .for themselves [and]. . .for the community.

CVI professionals are uniquely qualified to mitigate violence due to their compassion for the lived experiences of their clients, as well as their relatability andcredibility in their communities. However, the complexity of community-based firearm violence necessitates a robust skillset that enables CVI professionals to leverage their lived experience and structural praxis when engaging clients, providing resources, and addressing their underlying needs that require intervention. CVI practitioners recognize how cultivating more advanced skills in research, teaching, and advocacy will be beneficial for the CVI workforce and the communities they serve. While many organizations offer some form of ongoing training, CVI professionals report that such opportunities are inconsistent and often led by individuals who lack firsthand experience as frontline workers. This gap in training and professional development delivery undermines efforts to cultivate a sustainable infrastructure for CVI experts that reflects their daily realities.

There’s no [training] services that’s really stepped up to the degree that really relate to who we are, what we been through, and serves people with the same level of trauma.

Despite their eagerness to enhance their expertise, CVI professionals face funding barriers to accessing sufficient training and professional development opportunities. This funding disparity undermines individual capacity building and broader coalition development efforts across regions.

. . .I just would like to see more available funding for training, [a] significant amount of money to train our frontline workers [and] our leadership teams. . . [So] we can [go] to Sacramento and sit with. . .groups out there. . .advancing the work. Go to New York, Chicago, Texas, [go] to Baltimore. Have a conference. Have a retreat.

CVI professionals recognize the importance of building networks and cultivating a widespread infrastructure for CVIP that is well-informed and well-resourced. However, without adequate funding and support, they cannot fully realize their potential to innovate and lead within the field of CVIP. Expanding funding for comprehensive training programs that focus on the structural determinants of community-based firearm violence and professional development that advances the critical skills of CVI professionals are essential to tackle violence more effectively and sustainably. By investing in their education and capacity building, the field of CVIP can unearth new skills for strategy development and further cultivate the transformative potential of CVI practitioners in the fight against community-based firearm violence.

## Discussion

Illuminating the variation in structural competency held by CVI professionals provides insight into how well public health frameworks are being applied in CVIP programs and what information regarding the history of violence in America is being taught. For example, our findings reveal opportunities for CVI training to integrate frameworks such as Critical Race Theory (CRT). CRT provides a theoretical model for understanding the ubiquity and persistence of structural racism and its role in perpetuating harm (e.g., disproportionate resource divestment of marginalized communities), as well as the importance of counternarrative storytelling as an act of resistance (e.g., CVI professionals critically leveraging their insights and expertise).^37^

Furthermore, for programs to maintain fidelity in their evaluative infrastructure, in addition to broadening success measures to account for structural violence, they must ensure that CVI professionals receive standardized, comprehensive, and ongoing training to continue strengthening their theory of action. While it is evident that many CVI practitioners in this study understand how structural violence operates and its role in community-based firearm violence, they require opportunities to leverage this knowledge for more critical prevention efforts, such as researching the most effective violence interventions and advocating for stricter firearm policies at state and national levels. CVI professionals will unavoidably enter the field with varying levels of structural competency; however, until the evaluative and training infrastructures of CVIP account for the structural nature of community-based firearm violence, there will continue to be a disconnect between CVIP’s aims and its reported outcomes.

### Implications and Applications

Rigorous research and evaluation are integral to implementing and evaluating CVI strategies.^38^ Although CVI literature increasingly acknowledges the role of social and environmental factors in contributing to cycles of violence, contemporary program evaluations continue to rely on quantitative metrics that fail to capture the complexity of examining structurally grounded mediating factors to violence prevention, such as the knowledge, attitudes, and perspectives of the actors closest to those most at risk of violence engagement. CVI practitioners are vital in the fight against community-based firearm violence; their expertise, developed through their lived experiences, combined with their formal training as a CVI professional, uniquely qualifies them to engage with those most at risk of violence. The embodied narratives they have developed regarding the root causes of violence inform their theory of action as violence interrupters. Consequently, program evaluations must not only optimize how the contributions and expertise of CVI professionals are captured but also more specifically account for how their structural praxis informs their operation in the field.

Structural competency is imperative for any professional who works in violence prevention. CVI practitioners were uniquely identified as requiring *scholar-level* competency due to their specialized and ongoing proximity to individuals most at risk of violence; however, other practitioners such as educators, social workers, community leaders, and public service officials, would also benefit from structural competency.^21^ Imparting this knowledge, though, requires a standardized training and professional development curriculum guided by a structural framework. The curriculum should include hands-on training and engagement that prioritizes the examination of societal issues related to community-based firearm violence and researching and advocating for viable solutions to mitigate future harm. Doing so can equip practitioners in the field with the necessary skills, tools, and knowledge to guide their clients and align them with the appropriate resources by helping them identify and resist harmful traps in the system and reclaim the agency to navigate society.

The field of CVIP has begun developing academies for CVI practitioners and other key stakeholders in the field of violence prevention to provide ongoing, robust, and rigorous training and professional development. For example, the *CVI Leadership Academy (CVILA)*, developed by Dr. Chico Tillman with the Crime Lab at the University of Chicago, is the first of its kind training program for senior-level executives of CVIP programs. CVILA provides CVI leaders with five months of intensive, hands-on learning labs and lecture-based curriculum, facilitated by expert practitioners and academicians, focusing on strategic program management, leadership and staff development, data literacy and evaluation, and community engagement and advocacy. The five months of training culminate in a community capstone project where trainees are guided by an advisor to identify and examine an organizational or environmental issue that can be analytically examined and addressed through evidence-based solutions.^39^

The *CVILA* model incorporates a structural framework through project-based learning resulting in critical analysis and practical solutions to an identified issue within their community or the field. In the process, CVI professionals hone vital research and advocacy skills, which are significant domains within CVIP that necessitate further attention. Moreover, the Urban Peace Institute’s (UPI) *Peacemaker Academy* in Los Angeles focuses on gang intervention training for effective resource guidance, law enforcement training to improve community relations, and summer safety strategies to enhance community engagement activities; thereby further demonstrating proof of concept in developing comprehensive capacity-building curricula.^40^ Though the academies have different program objectives, their holistic approaches for addressing social and environmental factors (e.g., resource access and engagement with law enforcement) and doing so through critical observation and intervention, reflect how the integration of a structural framework acknowledges and garners opportunities to challenge the root causes of community-based firearm violence. The expansion of CVI leadership training academies, coupled with structurally grounded program evaluations that capture the complexities of direct violence engagement, advances the legitimacy, scale, and fidelity of CVIP efforts.

### Strengths and Limitations

While this analysis draws from a larger qualitative study exploring the perspectives of CVI professionals, it is the first to center their structural perspectives, specifically their understanding of the root causes of community-based firearm violence. The study captures insights from a multi-city cohort of CVI experts; however, the findings cannot account for all CVI professionals or programs.

The interview and focus group guides employed in this study were developed collaboratively with community partners and informed by the literature. Although these instruments were shared with practitioners to ensure clarity and relevance, they were not validated through a formal psychometric process. Rather, we align with qualitative research standards of trustworthiness,^41^ attending to credibility, dependability, and transferability. Credibility was enhanced through member checking, in which interpretations were shared with participants or relevant stakeholders to ensure their perspectives were accurately represented. Dependability and transferability were supported through an audit trail, which documented analytic decisions and coding processes in a clear, transparent manner that others could assess or replicate.^42^

We also acknowledge the role of researcher positionality in shaping the interpretation of qualitative data. Our team brought diverse identities and experiences to the research process, therefore, we employed systematic coding, analytic memos, and collaborative interpretation to mitigate individual bias. Though, as with all qualitative studies, subjectivity cannot be eliminated. Accordingly, this work should be considered in conversation with other studies examining the perspectives and expertise of CVI practitioners and those most at risk of experiencing community-based firearm violence.^23,43,44^

## Conclusion

This study highlights the variation in structural violence expertise among CVI practitioners and its implications for the training and evaluative domains of CVIP. Though many CVI professionals demonstrate a deep understanding of systemic harm, uneven levels of understanding expose gaps in training and professional development creating opportunities for the field to build greater capacity to address violence at its structural roots. Embedding a structural framework into training and evaluation is essential to ensure that CVI professionals are equipped to move beyond immediate intervention and toward sustainable transformation. Standardized, ongoing, and structurally grounded training can help practitioners guide clients through complex social environments, critically analyze systems, and advocate for policy change. As frontline actors, CVI practitioners possess the insight and credibility to disrupt cycles of harm, but without intentional investment in their professional development, the field risks misalignment between its goals and outcomes. Strengthening their structural expertise is key to building a more responsive, equitable, and sustainable CVIP infrastructure.

## Supplemental Material

sj-pdf-1-inq-10.1177_00469580251376234 – Supplemental material for Understanding Structural Violence in Community Violence Intervention (CVI): A Multi-City Qualitative Analysis of Practitioner Perspectives Supplemental material, sj-pdf-1-inq-10.1177_00469580251376234 for Understanding Structural Violence in Community Violence Intervention (CVI): A Multi-City Qualitative Analysis of Practitioner Perspectives by Asia S. Ivey, Julia J. Lund, Amanda J. Aubel and Shani A. L. Buggs in INQUIRY: The Journal of Health Care Organization, Provision, and Financing
