# Supplementary material for: Understanding Structural Violence in Community Violence Intervention (CVI): A Multi-City Qualitative Analysis of Practitioner Perspectives
Source: Inquiry. 2025 Sep 16;62:00469580251376234. doi: 10.1177/00469580251376234 (PMC12441253; doi:10.1177/00469580251376234)
Supplement: sj-pdf-1-inq-10.1177_00469580251376234 – Supplemental material for Understanding Structural Violence in Community Violence Intervention (CVI): A Multi-City Qualitative Analysis of Practitioner Perspectives [file sj-pdf-1-inq-10.1177_00469580251376234.pdf]

Table 1

Consolidated criteria for reporting qualitative studies (COREQ): 32-item checklist

No	Item	Guide questions/description	
**Domain 1: Research team and reflexivity**			
Personal Characteristics			
1.	Interviewer/facilitator	Which author/s conducted the interview or focus group?	Dr. Shani Buggs
2.	Credentials	What were the researcher's credentials? *E.g. PhD, MD*	PhD, MPH
3.	Occupation	What was their occupation at the time of the study?	Assistant Professor
4.	Gender	Was the researcher male or female?	Female
5.	Experience and training	What experience or training did the researcher have?	Shani Buggs is a health and public policy scholar with expertise engaging federal, state, and local officials, agency leaders, and organizations on comprehensive and community-driven violence intervention and prevention efforts. She uses qualitative, quantitative, and mixed-methods approaches to examine state-level firearm policies and local-level violence reduction initiatives.
Relationship with participants			
6.	Relationship established	Was a relationship established prior to study commencement?	Yes

7.	Participant knowledge of the interviewer	What did the participants know about the researcher? e.*g. personal goals, reasons for doing the research*	Participants were recruited through researcher's social network and prior connections to frontline violence prevention professionals in each of the study cities. They were told before the discussions what the study was about and given the opportunity to ask questions before, during, and after consent was granted.
8.	Interviewer characteristics	What characteristics were reported about the interviewer/facilitator? e.g. *Bias, assumptions, reasons and interests in the research topic*	Participants were told that the interviewer was interested in illuminating their perspectives and experiences in order to help inform future policy and practice related to gun violence prevention.
**Domain 2: study design**			
Theoretical framework			
9.	Methodological orientation and Theory	What methodological orientation was stated to underpin the study? *e.g. grounded theory, discourse analysis, ethnography, phenomenology, content analysis*	Grounded theory and phenomenology
Participant selection			
10.	Sampling	How were participants selected? *e.g. purposive, convenience, consecutive, snowball*	Purposive and convenience
11.	Method of approach	How were participants approached? e.*g. face-to-face, telephone, mail, email*	Face-to-face and email
12.	Sample size	How many participants were in the study?	45

13.	Non-participation	How many people refused to participate or dropped out? Reasons?	N/A
Setting			
14.	Setting of data collection	Where was the data collected? e.*g. home, clinic, workplace*	Participants' workplace locations and public settings chosen by participants.
15.	Presence of non-participants	Was anyone else present besides the participants and researchers?	No
16.	Description of sample	What are the important characteristics of the sample? *e.g. demographic data, date*	Please refer to manuscript. All were community violence intervention and prevention professionals with frontline expertise.
Data collection			
17.	Interview guide	Were questions, prompts, guides provided by the authors? Was it pilot tested?	Yes; No
18.	Repeat interviews	Were repeat interviews carried out? If yes, how many?	No
19.	Audio/visual recording	Did the research use audio or visual recording to collect the data?	Audio recording
20.	Field notes	Were field notes made during and/or after the interview or focus group?	Yes
21.	Duration	What was the duration of the interviews or focus group?	Approximately 1 hour
22.	Data saturation	Was data saturation discussed?	No
23.	Transcripts returned	Were transcripts returned to participants for comment and/or correction?	No
**Domain 3: analysis and findings**			
Data analysis			

24.	Number of data coders	How many data coders coded the data?	4
25.	Description of the coding tree	Did authors provide a description of the coding tree?	No
26.	Derivation of themes	Were themes identified in advance or derived from the data?	Both
27.	Software	What software, if applicable, was used to manage the data?	Rev.com and Dedoose
28.	Participant checking	Did participants provide feedback on the findings?	Yes
**Reporting**			
29.	Quotations presented	Were participant quotations presented to illustrate the themes / findings? Was each quotation identified? e.*g. participant number*	Yes
30.	Data and findings consistent	Was there consistency between the data presented and the findings?	Yes
31.	Clarity of major themes	Were major themes clearly presented in the findings?	Yes
32.	Clarity of minor themes	Is there a description of diverse cases or discussion of minor themes?	Yes
